# The Treatment of Fractures

**Published:** 1897-05

**Authors:** 


### Selections and Abstracts.

### THE TREATMENT OF FRACTURES.

Dr. G. G. Davis gives the following summary in a paper on "The Treatment of Fractures" in the *Annals of Surgery*.

1. It is my belief that massage and passive motion is not used to the extent that it should be in the treatment of fractures.

2. That immobility of the fractured ends favors quick union with little deformity.

3. That there are some cases in which, owing either to peculiarities of the fracture or the impaired constitution of the individual, the tendency to the formation of callus is marked. Motion in these tends to the formation of exuberant callus and deformity.

4. There are others in which bony union is unduly delayed; disturbance of the fractured ends in these hinders union.

5. It is wise to wait until the fractured parts are glued together, usually eight or ten days, before attempting any except the lightest massage, and any extensive passive motion after that time should be used carefully but diligently.

6. Passive motion and massage when first attempted should be of the most gentle character, and not so violent as to disturb the relation of the broken bones.

7. Any marked pain and inflammatory reaction following passive motion and massage is evidence that it has been too violent.

8. The limb should receive massage and manipulation at each inspection or change of dressing, often daily.

9. That in some cases it is advisable to administer such massage as is possible without removing the splints.

10. That persistent stiffness, particularly in fractures or injuries of the wrist, is often due to a rheumatoid affection locating itself in the injured region. Massage is valuable in the treatment of such.

11. Massage should be given to that part of a limb beyond the seat of fracture to preserve it in a normal condition.

12. Such dressings and methods of treatment should be adopted as will allow of the greatest use of massage and passive and active movements consistent with proper retention of the fragments.

